# Factors involved in nurses' responses to burnout: a grounded theory study

**DOI:** 10.1186/1472-6955-3-6

**Published:** 2004-11-13

**Authors:** Forough Rafii, Fatemeh Oskouie, Mansoure Nikravesh

**Affiliations:** 1 Faculty of Nursing and Midwifery, Iran University of Medical Sciences, Rasid Yasami st. Valiasr Ave. Tehran 19964, Iran

## Abstract

**Background:**

Intense and long-standing problems in burn centers in Tehran have led nurses to burnout. This phenomenon has provoked serious responses and has put the nurses, patients and the organization under pressure. The challenge for managers and nurse executives is to understand the factors which would reduce or increase the nurses' responses to burnout and develop delivery systems that promote positive adaptation and facilitate quality care. This study, as a part of more extensive research, aims to explore and describe the nurses' perceptions of the factors affecting their responses to burnout.

**Methods:**

Grounded theory was used as the method. Thirty- eight participants were recruited. Data were generated by unstructured interviews and 21 sessions of participant observations. Constant comparison was used for data analysis.

**Results:**

Nurses' and patients' personal characteristics and social support influenced nurses' responses to burnout. Personal characteristics of the nurses and patients, especially when interacting, had a more powerful effect. They altered emotional, attitudinal, behavioral and organizational responses to burnout and determined the kind of caring behavior. Social support had a palliative effect and altered emotional responses and some aspects of attitudinal responses.

**Conclusions:**

The powerful effect of positive personal characteristics and its sensitivity to long standing and intense organizational pressures suggests approaches to executing stress reduction programs and refreshing the nurses' morale by giving more importance to ethical aspects of caring. Moreover, regarding palliative effect of social support and its importance for the nurses' wellbeing, nurse executives are responsible for promoting a work environment that supports nurses and motivates them.

## Background

Working in a burn unit has been described as a stressful occupation [1]. Every nurse who cares for a burn victim knows that stress is a part of working in this field. Some authors have emphasized that these nurses experience dealing with self-inflicted burns, uncooperative patients, inter-staff conflicts and dying patients on a daily basis [2]. Unresolved job stress may results in emotional withdrawal and burnout [1]. Professional burnout has been defined as a syndrome manifested by emotional exhaustion, depersonalization, and reduced personal accomplishment [3]. Nurses who have worked in burn centers of Tehran have experienced burnout in comparison to nurses working in other areas. The main researcher's previous study of burnout and coping in burn centers of Tehran indicated that the majority of nurses had been experienced high levels of burnout [unpublished thesis]. The consequences of professional burnout for nurses are serious. It results in emotional withdrawal or indifference; reduces the limits of nurses' activity and their contact with patients [4]. Burnout results in a poor quality and quantity of nursing care and has negative effects on the most areas of personal, interpersonal and organizational performance [5].

While no health-care professional is immune to these pressures, there is evidence that suggests that areas of nursing particularly those areas we think of as critical care environments such as burn units, are often the most vulnerable to stress, and in need of much support [6,7]. Nurses in burn centers of Tehran are also vulnerable to burnout because these centers have many problems. The managers of the burn centers have not the authority for recruiting new nurses. Moreover, self-management of burn centers in Tehran, poverty of most of the burn victims and lack of supportive organizations, resulted in financial problems in burn centers. These in turn have resulted in intense staff shortages, a heavy workload, and low pay. These factors, in addition to inherent characteristics of burn centers have put nurses under a huge pressure and many times they have indicated that they haven't any motivation to work and they wish to leave burn centers as soon as possible. Lewis et al. had the same idea and concluded that the scope and intensity of problems nurses encounter in burn units indicate that they need psychiatric consultation [2].

However, regarding emotional, attitudinal, psychosomatic, behavioral and organizational responses of these nurses to burnout, it is vital to identify the factors that involve in their perception of burnout. Some authors also referred to these factors in burn centers [1] and other units or populations [8,9]. Nurses in burn centers of Tehran also pointed implicitly or explicitly to some factors that have played a role in their perceived stress and altered their responses to burnout.

The challenge for managers and nurse executives of burn centers is to understand the intervening factors and their impacts on these burn nurses' responses to burnout. As a result they can develop and promote delivery systems that support positive adaptation to stressors in burn centers, retain nurses and facilitate quality nursing care.

## Methods

In order to understand nurses' perceptions of factors modifying their responses to job stress and burnout, qualitative research adapted from the grounded theory method was chosen [10].

### Grounded theory

The value of using a qualitative research method such as grounded theory is embedded in the subjective and often emotional nature of care, stress and coping. As a descriptive study, the qualitative paradigm, with its emphasis on understanding factors modifying nurses' responses to job stress and burnout from the view point of practicing nurses themselves seemed logical. Grounded theory is a theory that is derived from data, systematically gathered and analyzed through the research process [10] The aim of grounded theory is to generate rather than verify theory [11]. The researcher's purpose in using grounded theory is to explain a phenomenon from within the social situation itself and to identify the inherent processes operating therein [12].

In effect, grounded theory is guided by simultaneous analysis. Both analysis and data collection inform each other. The analysis process is systematic and ends when new data no longer generate new insights. This has been also described as ' category saturation' [13,14].

### Pilot study

Five clinical nursing instructors participated in the pilot study. They were faculties of School of Nursing in Iran University of Medical Sciences (IUMS) and had been supervising nursing students in burn centers of Tehran for many years. Their age ranged from 40–48 years and had been working in burn centers for 7–14.5 years. The aim of pilot study was using the experiences of nursing instructors in the original study and reducing the informant and researcher bias in the interviews and participant observations [15].

The results of the study indicated that the staff of burn units felt drained, they haven't had any motivation or desire to care and they had been working purely for their pay. The findings were strongly indicative of the symptoms of burnout. It revealed that their behavior was representative of an indication of their professional dilemma. The pilot study also indicated that social support, patients' cooperation/motivation and the nurses' unique characteristics had been modified to alter the nurses' responses to burnout. Analysis of data from the original study was conducted keeping these findings in mind.

### Conduct of study

The research proposal was approved by the ethics committee of IUMS. Then permission was granted from the managers of two burn centers and their nursing administrators. Further permission and written consent was obtained from all who participated in this study.

### Data collection and sample

Following ethical approval, data was collected through tape- recorded, unstructured interviews. Initially data was collected in one center and analyzed. Then data gathering was initiated in the second center. There were 19 informants from the first center and 14 from the second center that participated (except 5 participants in pilot study). From this sample, 25 were nurses in different levels and positions and 8 were other members of burn team. The nurses' sample included 8 staff nurses, 8 licensed practical nurses, 2 nurses' aids, 3 head nurses, 2 supervisors, and 2 nursing administrators. Since nursing staff pointed to some issues concerning the burn team, the researcher interviewed one physician, one social worker, 2 physiotherapists, and 4 patients in the process of theoretical sampling.

Criterion for recruiting nursing participants was at least one year of experience in the burn center. Patients were selected according to their desire as well as their physical and psychological stability. Selecting patients occurred by consultation with head nurses.

Participants were 11 males and 22 females. The nursing staff participated were 19 females and 6 males. Twelve of the nursing staff had been working 2–3 shifts in burn centers or other hospitals as well as working in other jobs due to financial needs. Other demographic information of the nursing staff is displayed in Table 1.

**Table 1:** Demographic information of nursing staff.

**Demographic item**	**Mean**	**Range**	**SD**
Age of participants	39	23–52	± 9.5
Years of experience	17.5	1–29.5	± 10.5
Length of time at study hospital	12.85	1–29.5	± 10.9

Samples were recruited from all units of both centers. The first purposeful sample included 6 of nursing staff. Theoretical sampling was used after emerging the tentative theory. The basis for theoretical sampling was the questions which emerged during data analysis. At this stage the researcher interviewed nursing administrators and other members of the burn team. Theoretical sampling helped in verifying nursing staff's responses and credibility of categories and resulted in more conceptual density. No new data were emerged in the last two interviews; therefore data gathering by interviews were terminated.

### Interview process

Interviews were conducted in a private place with mutual agreement of the interviewer and interviewees. All interviews were completed by the main researcher. The duration of interviews ranged between 30–165 minutes. All the interviews were tape recorded except one. Some notes were taken during dialogues.

Unstructured interviews were conducted using a topic guide which has been drawn up by the researchers initial review of the literature related to the concepts of the subject of the study. This topic guide included the structure, process and outcome of care [16].

The following grand tour question guided the study:" please tell me about the nursing care in your unit". Subsequent questions were based on the participants' responses and demands of the emerging theory. Interviews were terminated when data redundancy occurred.

### Participant observation

In each center after the termination of interviews, participant observations were performed in all wards at morning, evening and night. 14 sessions of observation in the first center and 7 in the second center occurred. For this purpose the researcher informed the nursing administrators of her program. By selecting all the wards in all shifts there was no need for theoretical sampling in this stage (place and time) [10], but theoretical sampling of different situations were made in each ward or dressing room based on the questions which emerged during the interviews and observations.

Descriptive, focused and selective observations were occurred in a non- linear fashion. Theoretical sampling occurred during focused and selective observations. Some of the questions which guided the theoretical sampling were," is there any difference between nursing cares received by different patients?", "is nursing care different in a large or small ward?"

Prolonged engagement of the researcher in the field reduced the obtrusiveness. The level of participation varied from complete observation to participation in some activities. Some informal interview also occurred during the observations. Immediately after each session of observation field notes were completed systematically. Analysis of the field notes helped in determining contextual conditions and explaining variations in the nurses' responses in each context. This led to proposition of several hypotheses.

### Data analysis

Data collection, analysis and interpretation occurred simultaneously, in keeping with grounded theory methodology [10]. After each interview the transcript was manually transcribed by the main researcher onto a personal computer, providing an opportunity for identifying themes as the tape was transcribed(for the purpose of this paper, quotes from the participants were translated verbatim). Following transcription, a print- out was obtained and the tape replayed making notes onto the transcripts. Notes included comments about tone of voice, recurrent themes and the researcher's own initial thought and feelings about the nature and significance of the data. Field notes of each session of observations were also typed in double space and were analyzed. The transcripts were re- read and codes assigned to recurrent themes. This is known as "open coding", whereby the data are examined word by word and line by line [10], and codes were freely generated, often reflecting the words of the respondents themselves. For example the code "head nurse support" was given to the response:" relationship is heartfelt, perhaps I do many extra things because she is positive, she is supportive, she gives me motivation". The codes similar in meaning grouped in the same categories. Analytical tools include asking questions and making comparisons helped in finding the properties of each concept [10]. In axial coding, categories were related to their subcategories; coding was occurred around the axis of a category, linking categories at the level of properties and dimensions [10]. In this stage the structures of care were related to the processes. For example it indicated that which group of factors has contributed to the nurses' distancing from patients.

The process of integrating and refining the theory occurred in selective coding [10]. In this stage the core category" emergence of negative trends: nurses' responses to burnout" was identified. Selective sampling of literature related to job stress and burnout was very helpful. The core category linked other main categories (emotional, attitudinal, psychosomatic, behavioral, and organizational responses) and their subcategories. For the purpose of this article, the main categories and their subcategories are displayed in Table 2.

**Table 2:** Emergence of negative trends: nurses' responses to burnout.

**Main categories**	**Subcategories**
**Emotional responses**	Personal desperation
	Professional desperation
**Attitudinal responses**	Depersonalization
	Negativity
**Psychosomatic responses**	Physical attrition
	Psychological attrition
**Behavioral responses**	Intolerance
	Justification
**Organizational responses**	Perfunctory care
	Declining performance

### Data trustworthiness

The researchers accepted the perspective of Guba and Lincoln. They translated internal validity into credibility, external validity into transferability, reliability into dependability, and objectivity into confirm ability [17].

Credibility enhanced by the researchers' describing and interpreting her experiences. For this purpose the researcher kept a field journal in which she noted the content and the process of interactions, including reactions to various events. This journal became the record of relationships and provided material for reflection. Prolonged engagement and persistent observation helped to data credibility. In this way the process of data collection and analysis took 8 months. Data triangulation and method triangulation confirmed credibility [15]. Maximum variation sampling, participant observation and using published literature met this criterion. Furthermore, once the description of the phenomenon was complete, it was returned for verification to 4 participants of each center and they validated the descriptions. The original context described adequately, so that a judgment of transferability can be made by readers. The process of the study was audited for meeting dependability [18]. In doing so, student's supervisors and two other experts reviewed the process of the study and they arrived at a same conclusion.

Confirm ability requires one to show the way in which interpretations have been arrived at via the inquiry. In this study, confirm ability was established, because credibility, transferability and dependability were achieved [17]. The signposts indicating research decisions and influences were present throughout the study and the entire study functioned as an inquiry audit.

## Results

The findings related to factors which involved in the nurses' responses to burnout are presented in this article. Analysis and interpretation of data indicated that personal characteristics and social support have involved in the nurses' responses. These factors are presented in Table 3.

**Table 3:** Factors involve in nurses' responses to burnout.

**Personal characteristics**	Nurses' characteristics
	Patients' characteristics
**Social support**	Head nurse support
	Nursing administrator support
	Peer support

### Nurses' characteristics

Data from interviews and participant observations indicated that special personal characteristics and personality traits have involved in the nurses' emotional, attitudinal, behavioral, and organizational responses to burnout. Personal characteristics such as conscience, religious beliefs, personal philosophy, commitment, a sense of responsibility, and altruism facilitated caring behaviors. Nurses with these characteristics were more patient and empathetic. They were more cooperative and rarely justified their faults by fatigue, workload or staff shortage. Conscience, commitment, and religious beliefs such as fear of divine requital were the most prominent traits that modified the responses to burnout. One of participants stated:" God knows. I always feel it's me there on the bed. Sometimes a patient calls and I ignore, but I tell myself, what I expected if I were on this bed? I fear god and say to myself, his authority is great and whatever I do, I will see the reflection of my doings". Some of the participants pointed to interest and love in caring of burn victims. One of participants stated:" these patients are different from other ones. I have worked more than 18 years in general hospitals, I haven't worked more than 7 years in burn centers, but I think that was a blessing in disguise, I am glad because clinical work for a burn patient means love, means everything, believe me. I don't care the managers' behaviors, workload, nursing shortage and other deficiencies, because I love burn victims. Believe me". Many of the nursing staff, distanced from patients, they had immoral beliefs and demonstrated humiliation and reproach in their behaviors. They related these attitudes to fatigue, micro and macro conditions in burn centers, and loss of motivation; but participant observation indicated that, this isn't the case for all the nurses. Nurses, who had been known as good nurses, were very calm and intimate with their patients and focused on the patients' needs. The researcher wrote in one of her field notes:" she is very calm and speaks with compassion. She makes jokes and patients are relaxed with her. She follows the principles and procedures more strictly than others". Nurses in all levels were under pressure of workload, low pay, staff shortage, environmental conditions of burn units and other structural inhibitors, but as the excerpts of interviews indicated, appraisal of these inhibitors was different in the presence of specific personal characteristics.

Data indicated that in some instances, when there were a number of inhibitors and they were long stay, even positive personal characteristics couldn't work. This was often the case in infectious dressing rooms and busy wards. The worst kinds of treating patients were seen in these places. It seemed that the inhibitory factors which are persistent and too frequent interact with personal characteristics and finally overcome the positive characteristics. One of participants stated:" patients expect to receive care, expect a friendly encounter which does not happen. To tell the truth, some days I am excessively distressed and tired that I don't have the patience to answer the patient' questions and concerns. I emotionally can not do what I really want to do on a daily basis for the patients. The reason is persistent day and night problems occurring with this job". This process is displayed in Table 4.

**Table 4:** Interrelationships between personal characteristics, inhibitory factors and caring behavior in burnout.

**Frequency and intensity of inhibitory factors**	**Positive personal characteristics**	**Caring behavior**
High	Defeat	Deteriorates
Low	Overcome	Improves

### Patients' characteristics

Data strongly indicated that nurses' appraisal of the patients' characteristics have influenced some aspects of their attitudinal and behavioral responses. When the appraisal was positive, the relationships improved, and when it was negative, relationships deteriorated. Positive appraisal occurred often when the patients were cooperative and motivated for recovery and in cases where they had an advantage of high socio- economic class, cultural and educational levels, or whenever they stimulated the nurses' senses of compassion and pettiness. Negative appraisal occurred often when the patients were from lower socio- cultural levels, addicts or there was a possibility of having acquired immune deficiency syndrome (AIDS) or hepatitis.

The first group was treated kindly and more respectfully. The use of humiliating words and reproach towards them diminished and as a result the aggressive behavior and physical withdrawal less occurred. One of participants stated:" ...I am more supportive and compassionate towards children, those who are very alone, who have no one to love and care for them, those who have committed self-inflicted burns, a woman whose husband caused her to burn herself and doesn't have any one to support her. In many occasions I have even paid them to buy juice from outside the hospital. I feel that these patients are needy".

The second group was treated very unethical. They encountered a humiliating, reproaching, and aggressive behavior. One of participants stated:" ...he fights when I am dressing him, he pulls his hand and leg, he isn't cooperative, and he has no class. They drain all my energy to the point that I don't want to talk to them. I think they are mentally retarded. They keep still when I shout at them just the way that the children act. I tell them I'll pull your ear, and I'll beat you up. This is the behavior that has worked with them ".

Moreover patients with extensive burns, whose survival was an improbable event, not only were badly treated, but also were sometimes ignored and received poor care. In other words, they received only those treatments which had been ordered by physicians to prevent being reprimanded by supervisors. One of the participants justified herself and stated:" if you want the truth, a patient with 90% burns can not benefit from tetracycline ointment, but it's there in his order, I prefer to spend my time with a patient who has a better chance of surviving. Right or wrong I don't apply the ointment, because I can spend that time for a patient who will survive". We can conclude that burnout has made nurses to modify their caring behaviors to fit the different type of patients they care for.

### Interaction between nurses and patients' characteristics

As described later, nurse's and patient's characteristics modified the nurse's responses to burnout and altered caring behaviors. More analysis and interpreting of data indicated that interaction between these two variables resulted in a more powerful combination that alters responses to burnout and identifies the kind of caring behavior. This process is displayed in table 5. It is important to mention that the meaning of patient's characteristics in this study is the nurse's appraisal or perception of these characteristics and nurse is clinical nursing staff in different levels.

**Table 5:** Interrelationships between nurses and patient's characteristics and caring behavior in burnout.

**Nurse's characteristics**	**Patient's characteristics**	
	**+**	**-**
**+**	Naturally good behavior	Relatively good behavior related to ethical aspects- sometimes non-ethical
**-**	Relatively good behavior related to inhibition of non-ethical aspects	Bad behavior related to the opportunity for emerging non-ethical aspects

Table 5 indicates that when both nurse's characteristics and her/ his appraisal of patient's characteristics are positive, then the nurse's caring behavior is naturally effective and efficient. In this case, patient is treated respectfully, there is an empathetic behavior, and nurses spend more time with her/his clients to value their emotional needs. When the nurse's characteristics are positive and her/ his perception or appraisal of the patient's characteristics is negative the nurse doesn't have a natural empathetic behavior. She thinks that she has to be good and behave well because of her beliefs; therefore she demonstrates a good behavior. Sometimes when the patient has been perceived as having a negative outburst, from the nurse's point of view, she/he has a very negative attitude towards the patient. This will cause ethical issues and misbehavior by the nurse. The good behavior occurs when the nurse's characteristics are negative but the appraisal of the patient's characteristics is positive. In this case, the patient's characteristics do not permit for emergence of negative characteristics of the nurse; therefore an ethical/respectful and caring behavior will result. At times when nurse's characteristics are negative and her/his perception of the patient's characteristics is also negative, non-ethical behaviors find a good opportunity to emerge. In this situation the patient encounters the worst behavior. Humiliation is intense, physical withdrawal is often seen and aggressive behavior is routine.

One of participants stated: "I take care of some patients with love and conscience and take care of the other patients only with conscience and some nurses doesn't have love at all, I take care of silent, calm and lonely patients better, I perform routine care for the other". Another participant also stated:" it seems I give positive energy to the patients to whom I am more interested and take care of them with more love. I've seen that they respond better to therapeutic measures". Therefore interaction between nurses and patients' characteristics has a very powerful effect on the nurses' responses to burnout and determines their nature of caring behavior.

### Social support

Data from interviews and participant observations strongly suggested that social support influences the nurses' responses to burnout.

Supportive behavior of head nurses, nursing administrators and coworkers modified the nurses' responses. Among these, head nurse's the support was the most effective factor. Nurses believed that they do not have any motivation or desire to perform well when they are not supported well. One of participants stated:" we have a very close loving relationship with the head nurses who are supportive. In that situation I do many things for her. Her supportive attitude and caring/positive attitude helps me a lot. Do you understand?" another participant believed that he couldn't endure if the nursing administrator weren't supportive. He stated:" I have seen that she is doing a good job. I have been so stressed at times that I have thought of quitting. The nursing administrator has changed my mind during those stressful moments by being caring, loving and supportive and I have decided to stay in spite of low pay and benefits." Support from peers also modified the nurses' responses. They could tolerate more with the support of their coworkers. One of participants stated:" by god, I like every single one of them. It seems like we live together seven hours a day (major part of our day). We are so familiar with each other's character and behavior patterns. You may not believe this, these relationships has been very helpful and caused us to have a very strong unit. We care for each other in times of weakness, illness or pressure".

It is worthy of mention that the effect of social support on the nurses' responses to burnout was not as powerful as the nurses' and patients' characteristics. Social support influenced the emotional and in some cases the attitudinal responses, but it didn't have enough power to alter the organizational and behavioral responses, therefore it didn't change caring behaviors significantly. Moreover, there were not any data indicative of the modifying effect of the factors proposed in this article on the psycho- somatic responses to burnout.

## Discussion

Findings of this study indicated that nurses' and patients' characteristics and social support modified the nurses' responses to burnout. These factors altered the nurses' perceptions of the inhibitory factors; in other words they could have a more positive appraisal and this in turn modified their responses.

Lazarus and Folkman (1988) proposed that the initiative of behavioral manifestations is a transactional appraisal which is important for the person's wellbeing and betrays the confrontation as noxious, useful, threatening or requiring struggle [19]. Moreover Lazarus (1976) believed that personal variables including values, beliefs, commitment and a sense of control over the environment are modifying factors that influence a person's cognitive appraisal [20]. In this study, conscience, religious beliefs and commitment were the most prominent characteristics that modified the nurses' responses to burnout. Nurses with positive characteristics had a non- threatening evaluation of their confrontations, therefore they cared better for their patients. Garrett and MC Daniel (2001) in their study concluded that a nurse's perception of the environment is more a function of personality than education or experience [8]. Conscience and commitment were of the most prominent characteristics that modified nurses' responses to burnout, and were related to the caring behavior. Focusing on caring attitudes, Roach (1987) also proposed that caring behavior in nursing is manifested through the five 'C ' attributes: compassion, competence, confidence, conscience and commitment [21]. Stuart and Sundeen (1987) referred commitment as representative of whatever important for the person. It includes decisions the person considers as necessary in his life and it can direct people to (or far from) the conditions which could be threatening, noxious or probably useful [22]. Participants pointed mostly to religious beliefs as a modifying factor. Stuart and Sundeen (1987) also concluded that spiritual beliefs can essentially reduce stress and influence on the persons' potential coping capabilities [22].

In this study, when inhibitory factors were persistent and frequent, even positive personal characteristics defeated. Selye (1976) concluded that the influence of stressors on a person depends on the number of stressors that must be confronted in a time, the duration of confrontation and existence of previous experience with the same stressors [23]. This happened more in infectious dressing rooms, where nurses had the closest longest contact with the bare bodies of burn victims. There were only one dressing room with 18–27 patients per each day for dressing and some of nurses had been there for more than 28 years.

Patients with different characteristics treated differently. Nurses changed their caring behavior with different patients. It seemed that they didn't have enough emotional and physical energy and motivation for caring for all patients; but some patients stimulated their emotions and gave them the needed positive energy for caring. In other words, patients' characteristics could both reduce and intensify the nurses' responses to burnout. When these characteristics appraised as positive, caring behaviors improved, and when it was negative, the worst kind of behaviors occurred. Maslach (1982) in congruence with this conclusion believed that there is little evidence that caring is a uniform state [24], and Benner and Wrubel (1984) concluded that it's not clear that this is because the caring affect is depleted, or the nurse's personal needs for emotional protection take precedence over the human caring for others. They also believed that physical exhaustion may reduce the nurse's ability to continue to provide care [25].

Patients with extensive burns received the worst kind or care. Nurses stated that caring for these patients is futile. Data implied that caring for these patients have been incongruous with the nurses' values. Meltzer and Huckabay (2004) in their study of the relationship between critical care nurses' perceptions of futile care and its effect on burnout, concluded that feeling of emotional exhaustion in these nurses was highly influenced by the frequency with which nurses were involved in life- sustaining interventions that conflicted with the nurses' values and standards in term of what the nurses thought are ethically appropriate and could result in improvement in a patient's condition and outcome [26].

It was the interaction between nurses' and patients' characteristics that identified the caring behavior. Some authors have found according to their clinical practice that nurses have the ability to adjust their approach and their style of interaction with different patients. These authors proposed that they not only alter the nature of dialogue and the tone of voice to meet each patient's needs, but also adjust their affective response. They pointed that delineation of these behaviors would be a significant contribution, yet to date these styles of care have not been explored [27]. In this study some patients received a natural care, but others faced with an ethical and in some instances a non- ethical care. Natural care occurred spontaneously and without thinking, but ethical care happened thoughtfully by the mediation of the patients and/ or nurses positive characteristics. Typically non- ethical cares was thoughtful, but in this case both the nurses and patients characteristics were negative and the appraisal was too threatening. In her discussion of caring, Noddings (1984) also distinguished between natural caring and ethical caring. According to Noddings, natural caring comes from a remembrance of being cared for, whereas ethical caring " is an active relation between my actual self and a vision of my ideal self as one- caring and cared- for" [28]. Other authors also proposed that nurses may care naturally or they may care out of a desire to be a good nurse [29].

Social support from head nurses, nursing administrators and peers made nurses to endure and tolerate in the face of problems. Lazarus (1976) proposed that the resources reducing the potential harm could be found in the environment and essentially in others who indicated one can rely on them [20]. Social support didn't have enough power to modify behavioral and organizational responses and it could only change emotional and some aspects of attitudinal responses. Therefore caring behaviors didn't change. Garrett and MC Daniel (2001) also concluded that other people in the work setting like supervisors and peers might limit depersonalization and emotional exhaustion [8].

## Conclusions

This study as a part of more extensive research (PhD dissertation) identified the most important factors that intervened in the nurses' responses to burnout in burn centers of Tehran. Nurses had responded to burnout. These responses included emotional, attitudinal, psychosomatic, behavioral and organizational. This part of study indicated that the nurses and patients characteristics and interaction between these two factors had a very powerful effect on the responses and determined the kind of caring behavior. Moreover social support from managers (e.g. head nurses and nursing administrators) and peers modified some of the nurses' responses to burnout.

The influence of positive personal characteristics, especially conscience, religious beliefs, philosophy, commitment, a sense of responsibility and altruism on the nurses' responses to burnout, the finding that, long lasting and persistent problems in the work setting can deteriorate even the personal characteristics, and regarding the numerous problems in burn centers of Tehran, there is an urgent need for helping the nurses. We suggest that due to the intense staff shortage in these centers, the managers try to keep these nurses. The only way they can do this is by using stress reduction programs. Data strongly indicated that these nurses need to rest periodically to preserve energy and to refresh their morale. Moreover, giving importance to moral and ethical aspects of care by managers could be helpful and motivating. Changing burn patients' inherent characteristics and their other characteristics such as poverty and socio- cultural level is not a possible alternative. Promoting nurses' morale is possible and must be done promptly if we want our burn survivors receive at least an ethical effective care.

Social support made the problems tolerable. Therefore we recommend nurse executives in burn centers of Tehran promote a work environment that help to decrease the perception of pressures and increase perceptions of social support. The best solution is of course eliminating the micro and macro conditions which overshadow the nurses' responses and caring behaviors.

This grounded theory created several hypotheses in this stage. Nurses with specific traits were more resistant to burnout and were more caring. It is suggestive of conducting a quantitative research to test the relationship between personality traits, burnout and caring behaviors. Differences in caring behavior for different patients were related to the influence of burnout on the nurses' personality traits and appraisal of patients' characteristics. It was a very new finding that needs to be investigated in more detail.

The dramatic effect of social support on the nurse' perceptions of pressures in burn centers of Tehran is suggesting of identifying the relationship between social support and the level of burnout the nurses experience in these burn centers.

However the nature of a qualitative research like this, limits it's generalizability; therefore we suggest conducting more qualitative research in other critical care units to support these findings.

Application of the findings of this study and conducting the suggested studies would help the managers of burn centers to enhance an environment conductive to morale, promote natural and ethical care, refresh the nurses' positive emotions and facilitate a supportive environment for their nurses.

## Competing interests

The author(s) declare that they have no competing interests.

## Authors' contributions

FR initiated and designed the research, collected and analyzed the data and wrote the paper. FO was the main supervisor, helped in analysis, and revised and edited the drafts. MN was co- supervisor and revised the drafts.

## Pre-publication history

The pre-publication history for this paper can be accessed here:


